# NADPH Oxidase-Driven Phagocyte Recruitment Controls *Candida albicans* Filamentous Growth and Prevents Mortality

**DOI:** 10.1371/journal.ppat.1003634

**Published:** 2013-10-03

**Authors:** Kimberly M. Brothers, Remi L. Gratacap, Sarah E. Barker, Zachary R. Newman, Ashley Norum, Robert T. Wheeler

**Affiliations:** 1 Department of Molecular and Biomedical Sciences, University of Maine, Orono, Maine, United States of America; 2 Graduate School of Biomedical Sciences and Engineering, University of Maine, Orono, Maine, United States of America; University of Birmingham, United Kingdom

## Abstract

*Candida albicans* is a human commensal and clinically important fungal pathogen that grows as both yeast and hyphal forms during human, mouse and zebrafish infection. Reactive oxygen species (ROS) produced by NADPH oxidases play diverse roles in immunity, including their long-appreciated function as microbicidal oxidants. Here we demonstrate a non-traditional mechanistic role of NADPH oxidase in promoting phagocyte chemotaxis and intracellular containment of fungi to limit filamentous growth. We exploit the transparent zebrafish model to show that failed NADPH oxidase-dependent phagocyte recruitment to *C. albicans* in the first four hours post-infection permits fungi to germinate extracellularly and kill the host. We combine chemical and genetic tools with high-resolution time-lapse microscopy to implicate both phagocyte oxidase and dual-specific oxidase in recruitment, suggesting that both myeloid and non-myeloid cells promote chemotaxis. We show that early non-invasive imaging provides a robust tool for prognosis, strongly connecting effective early immune response with survival. Finally, we demonstrate a new role of a key regulator of the yeast-to-hyphal switching program in phagocyte-mediated containment, suggesting that there are species-specific methods for modulation of NADPH oxidase-independent immune responses. These novel links between ROS-driven chemotaxis and fungal dimorphism expand our view of a key host defense mechanism and have important implications for pathogenesis.

## Author Summary

Over 45 years ago chronic granulomatous disease (CGD) was ascribed to a failure of neutrophils to mount a respiratory burst, and it is now known to result from primary genetic deficiencies in the phagocyte NADPH oxidase complex. Recent work suggests that reactive oxygen species produced by NADPH oxidases have other important functions as diverse as maturing hormones and promoting protein kinase signal transduction. *Candida albicans* is an opportunistic pathogen that preys on immunocompromised patients to cause lethal candidemia. We used the transparent zebrafish larva to describe a novel function of both phagocyte oxidase and dual-specific NADPH oxidase in directing phagocyte recruitment to *C. albicans* infection foci. We demonstrate that NADPH oxidase-dependent attraction of neutrophils and macrophages is instrumental in effective containment of yeast within phagocytes, which prevents the yeast-to-hyphal morphogenetic switch and limits mortality. Remarkably, when the fungal morphogenetic switch is prevented by mutation, NADPH oxidase activity is no longer required for effective fungal containment. Our study suggests that defects in CGD may extend beyond reduced microbial killing by superoxide to include impairment of chemotaxis, and provide a basis for exploring this alternative function in mammals.

## Introduction

*Candida albicans* is a ubiquitous commensal fungus and a clinically important opportunistic pathogen of humans. *C. albicans* is pleomorphic and grows in both yeast and filamentous forms, permitting growth in different environments, tissue invasion, dissemination and immune evasion [1]–[3]. Dimorphic switching is governed by myriad signals *in vitro* and is co-regulated with virulence factors; the links between dimorphism and pathogenesis *in vivo* are further complicated by the complexity of signals and the potential for immune control of differentiation [4]–[6].

Immunodeficiencies centered on either the innate or adaptive immune systems predispose for a number of opportunistic fungal infections with *Candida spp.* [7], [8]. Lack of phagocyte NADPH oxidase (Phox) components causes chronic granulomatous disease (CGD), a rare immunodeficiency associated with susceptibility to bacterial and fungal pathogens [9]. Over 45 years ago, the specific cellular defect in CGD was determined to be an inability of CGD leukocytes to mount a respiratory burst and kill microbes upon *in vitro* stimulation, yet this may not explain why CGD patients suffer from symptoms beyond susceptibility to acute infection, such as hyperinflammation and B-cell deficits [9]–[11]. Reactive oxygen species (ROS) produced during the respiratory burst are highly toxic to pathogens *in vitro* [12], [13], but it is now appreciated that ROS also impact many signaling pathways and cellular processes [9], [14]. Notably, both the phagocyte oxidase Phox and the dual-specific NADPH oxidase (Duox) have been implicated in promoting chemotaxis to specific stimuli and/or sites of inflammation, although it is not clear if either has a role in phagocyte recruitment to sites of infection [15]–[19].

NADPH oxidases are important for immunity to many pathogens, although their roles in protection against *C. albicans* are not clear-cut. Most *in vitro* experiments suggest Phox is important for killing *C. albicans*, and CGD mice are more susceptible to candidemia in the tail vein injection model [20]. Other work suggests a more nuanced role for Phox, as candidemia is a rare cause of death in CGD patients [7], [21], Phox is not absolutely required for control of infection in the mouse [22], and other phagocyte weapons can contain *C. albicans* both *in vivo* and *in vitro* [23]–[26]. The nematode model of mucosal candidiasis suggests that Duox can also play an important role in protection, although this has yet to be tested in mammals [27]. Current *in vitro* and *in vivo* models have not yet integrated these disparate data to explain the *in vivo* role(s) of NADPH oxidases in control of candidiasis.

The emerging larval zebrafish model provides a unique and powerful platform to discern how the *in vitro* activities of pleiotropic molecules such as ROS translate into *in vivo* roles during infection [28]–[30]. We recently showed that a larval model of disseminated candidiasis shares key aspects of mammalian disease [31]. We performed extended intravital imaging of live zebrafish to show that macrophages can inhibit germination of yeast into hyphae *in vivo*. Additionally, we found that the phagocyte oxidase is important in limiting filamentous growth *in vivo* [31]. Here we link these two observations to show that NADPH oxidase-dependent recruitment of phagocytes limits filamentous growth because it ensures that *C. albicans* is phagocytosed efficiently and is thus prevented from germination. We demonstrate that both Phox and Duox are required for efficient phagocyte recruitment, phagocytosis, limiting filamentous growth, and survival. We find that early immune recruitment is a strong and reliable indicator of eventual infection clearance. We also implicate the *EDT1*-dependent dimorphic switching pathway in modulating both fungal containment and virulence of extracellular fungi. These data identify a new dimension of NADPH oxidase-mediated immunity that strongly impacts fungal dimorphism in the host setting.

## Results

### Phagocytosis blocks germination independently of NADPH oxidase activity

We recently provided the first demonstration of an *in vivo* role for the phagocyte NADPH oxidase in limiting filamentous growth of *C. albicans* [31]. Here we sought to determine mechanistically how NADPH oxidase activity limits filamentation and susceptibility to infection. Traditionally, the most important role for NADPH oxidase in immunity has been ascribed to its ability to create reactive oxygen species that directly damage or kill microbes [13]. In fact, there is significant oxidative stress experienced by *C. albicans* when attacked by neutrophils or macrophages *in vitro* [32], [33]. Therefore, we first hypothesized that NADPH oxidase-derived oxidants in the phagosome might damage *C. albicans* and block intracellular germination in this infection to limit filamentous growth. To determine if fungal cells were under oxidative attack *in vivo*, we used the OxYellow-T oxidative stress reporter strain. This strain has the oxidative stress-induced *CTA1* promoter driving EGFP expression and the constitutive *ENO1* promoter driving dTomato expression. Using this strain we find oxidative stress at 24 hpi but not at 4 hpi in control morphants, whereas there is no detectable oxidative stress at either 4 or 24 hpi in phagoctye oxidase morphants (Fig. S1), and using a similar strain we have previously published that there is no detectable oxidative stress at 6 hpi in this model [31]. We also found no activation of the respiratory burst within phagocytes, as observable upon incubation of live infected fish with H_2_DCF-DA (Fig. S2), a cell-penetrating molecule that diffuses well into live zebrafish and fluoresces upon oxidation [31], [34]. Phagocyte oxidase-produced ROS have been demonstrated to drive localization of the autophagy reporter protein LC3 to the membrane of yeast-containing phagosomes in a process referred to as LC3-associate phagocytosis [35], [36]. To determine if a similar process occurs *in vivo* in zebrafish, we examined the localization of a GFP-LC3 fusion protein in phagocytes containing *C. albicans*. We used a transgenic line of zebrafish for which GFP-LC3 localization has been shown to report on autophagic activity [37]. In contrast to previously reported *in vitro* findings, we found very few phagosomes with GFP-LC3 localized to the phagosomal membrane *in vivo*, suggesting that ROS-mediated LC3 localization plays a less important role in this *in vivo* model than has been demonstrated *in vitro* (Fig. S3). Further, two treatments recently shown by Huang et al. [35] to strongly inhibit phagosomal LC3 localization *in vitro*—blockade of NADPH oxidase activity with the pan-NADPH oxidase inhibitor diphenyleneiodonium (DPI) and treatment with the anti-oxidant α-tocopherol—mildly reduced but did not significantly affect GFP-LC3 localization to phagosomes. Taken together, these data are not consistent with the idea that NADPH oxidase acts early to produce respiratory burst-derived oxidants that damage the fungi or traffic it to autophagosomes and thereby block filamentous growth.

**Figure 1:**
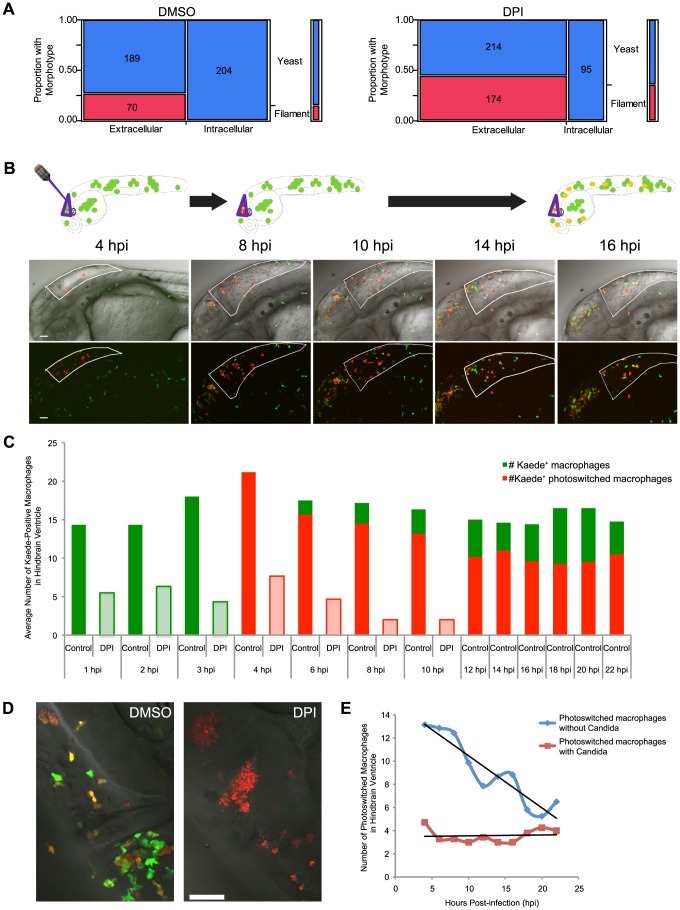
Photoswitching time-lapse shows phagocytosis block of germination is long-lasting and NADPH oxidase-independent. (A) CAF2-dTomato-infected fish were treated with DMSO or DPI and scored at 4 hpi for fungal morphotype and internalization. Filamentous growth is only seen extracellularly, and this difference is highly significant (p<0.001) in both DMSO (n = 11) and DPI (n = 12), as measured by Fisher's exact test. Data are pooled from three independent experiments. (B–E) CAF2-dTomato-infected Tg(*mpeg1:GAL4/UAS:Kaede*) fish were treated with DMSO (n = 11) or DPI (n = 7) from two hours pre-infection and throughout imaging. At 4 hpi, only macrophages at the infection site in the hindbrain were photoswitched green-to-red by exposure to 405 nm laser light. Photoswitched fish were imaged every two hours for an additional 18 hours. (B) Representative images of macrophage movement during the time-lapse, with a schematic above depicting the movement of macrophages out of the hindbrain and acquisition of yellow color as fresh green-fluorescent Kaede protein is produced. Scale bar = 50 µm. (C) Representative images of DMSO- (left) and DPI-treated (right) fish at 18 hpi, showing filamentous growth in the DPI-treated but not the control fish. Scale bar = 50 µm. (D) Macrophages at the site of infection were enumerated at each time-point. Green and red bars represent green-fluorescent (native Kaede) and red or yellow-fluorescent (photoswitched Kaede). Solid bars represent averages from control fish and lightly shaded bars from DPI-treated fish. Although all the infected, DPI-treated, fish died by 18 hpi, uninfected, DPI-treated, fish do not die due to this treatment alone (data not shown and Fig. 5D). (E) Movement of photoswitched macrophages from the hindbrain was tracked for two classes of Kaede+ macrophages: those that had internalized fungi and those that did not phagocytose fungi. A best-fit line for each population shows that half of those that did not phagocytose fungi have left by approximately 18 hpi, whereas there is no appreciable emigration of macrophages with internalized yeast from the hindbrain in this time. (D–E) Shown are the averages per fish pooled from three independent experiments of at least two fish per group.

Nevertheless, to determine if blockade of NADPH oxidase permitted germination of *C. albicans* within phagocytes *in vivo*, we examined the fungal morphotypes at 4 hpi with and without the pan-NADPH oxidase inhibitor DPI. Regardless of NADPH oxidase activity, there was a striking difference in morphotype between intracellular yeast and extracellular filamentous growth, with filamentous cells found only outside of phagocytes (Fig. 1A). This difference in intra- vs. extracellular fungal morphology is statistically significant in both vehicle- and DPI-treated fish (p<0.0001 by Fisher's exact test). Furthermore, there was no intracellular germination even upon blockade with DPI (0/204 for DMSO and 0/95 for DPI). Thus, in contrast to our expectations, inhibition of NADPH oxidase did not permit germination and filamentous growth within phagocytes early during infection. Instead, while some extracellular fungi switch morphotype and grow as filaments, phagocytosis can block germination even without NADPH oxidase activity. This suggests that there are other host immune mechanisms besides NADPH oxidase that can control *C. albicans* growth within phagocytes.

Because time is a crucial axis of disease progression, we extended these studies to follow the fate of internalized *C. albicans* beyond 4 hpi to identify any later roles for NADPH oxidase in limiting germination. To determine if phagocytes continue to control filamentous growth of internalized yeast up to 22 hpi, we took advantage of the Tg(*mpeg1:GAL4/UAS:Kaede*) photoswitchable macrophage line [38]. We photoswitched macrophages in the hindbrain ventricle of representative fish at 4 hpi and followed them by time lapse every 2 hours until 22 hpi (Fig. 1B). Again, we found that internalized *C. albicans* yeast did not germinate within macrophages or neutrophils for the duration of the time-lapse, even in the presence of DPI. In three independent experiments, a total of 279 fungi were followed in DMSO-treated fish (217 inside macrophages and 62 within neutrophils) while in DPI-treated fish with limited engulfment, a total of 50 internalized fungi were followed (26 within macrophages and 24 within neutrophils). Control experiments suggest that the lack of germination is not due to photoactivation itself or imaging-induced inhibition of filamentation. Specifically, there is no inhibition of filamentous growth by frequent imaging in both green and red channels over the first six hours of infection and there is no clear defect in pathogenesis or immunity upon photoactivation (Fig. S4). The lack of intracellular germination in these extended time-lapse experiments suggests that macrophages and neutrophils remain effective in suppressing filamentous growth of internalized fungi, even when NADPH oxidase activity is blocked.

### Long-term macrophage flux at the infection site is NADPH oxidase-dependent and related to fungal engulfment

Current *in vivo* models have not permitted extended assessment of *Candida*-phagocyte interactions at the infection site to characterize the dynamics of phagocyte migration. To examine the role of NADPH oxidase activity in controlling immigration and emigration of macrophages, we analyzed time-lapse experiments performed by photoswitching Kaede-expressing macrophages at the infection site at 4 hpi. We categorized the photoswitched and non-switched macrophage populations at the site of infection in the hindbrain ventricle between 4 hpi and 22 hpi. In control fish, about half of the photoswitched (red) macrophages left the hindbrain within 12 hours but were replaced by new (green) macrophages from outside of the infection site (Fig. 1C). However, in fish treated continuously with DPI there was migration away from the hindbrain but no replacement with new macrophages, leading to uncontrolled growth of the *C. albicans* (Fig. 1D). Importantly, control uninfected fish treated continuously with DPI suffered no ill effects. This suggests that the defects in immune infiltration associated with blockade of NADPH oxidase extend past 4 hpi and, if anything, are more severe when DPI treatment is continued to later times post-infection. Furthermore, these experiments document for the first time the dramatic flux of phagocytes to and from the infection site for hours post-infection.

In mammals, macrophages are heterogeneous in function and can differentiate upon stimulation to promote diverse host responses, although it has not been possible to examine the dynamic roles of different subtypes in the context of *C. albicans* infection [39]–[41]. Because our transparent model offers a unique tool to identify differential roles of individual phagocytes during *C. albicans* infection, we were able to quantify the divergent kinetics of egress from the hindbrain in two macrophage populations. We found that macrophages that internalize yeast tend not to move away from the site of infection within the first 22 hpi, while most macrophages that do not engulf fungi move away from the infection site during this time. Approximately 50% of non-phagocytic macrophages leave the hindbrain by 12 hours after photoswitching, while there is no bulk emigration of phagocytic macrophages within 18 hours after photoswitching (Fig. 1E). This suggests that phagocytosis of *C. albicans* is associated with reduced movement from the infection site.

### NADPH oxidase is required for neutrophil and macrophage chemotaxis to the site of *C. albicans* infection

**Figure 2:**
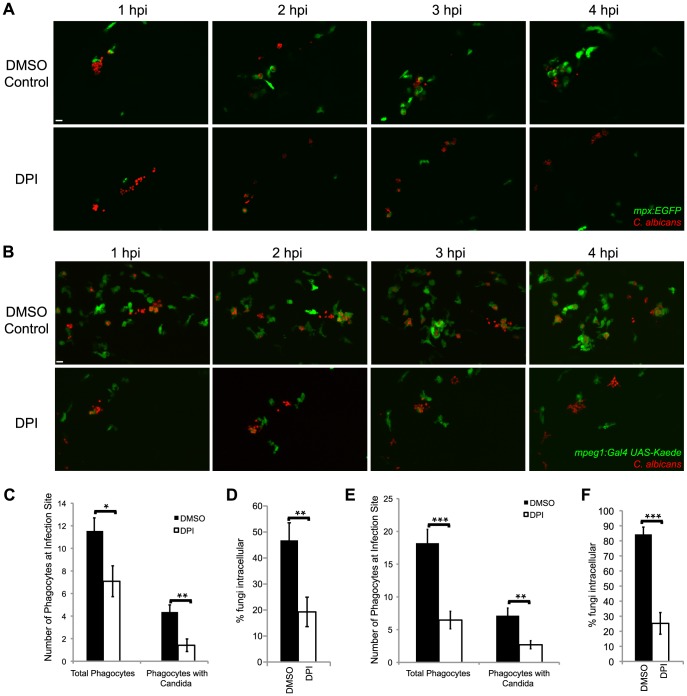
NADPH oxidase is required for efficient phagocyte recruitment and containment of *C. albicans*. (A–F) CAF2-dTomato *C. albicans* or were microinjected into the hindbrain ventricle of (A) Tg*(mpx:GFP)^i114^* (n = 11) or (B) Tg(*mpeg1:GAL4/UAS:Kaede*) (n = 15) prim25 stage larvae. Fish were treated with DMSO (vehicle) or DPI from two hours prior to infection to 4 hpi and imaged from 0 to 4 hpi by confocal microscopy. (A–B) Images represent at least three independent experiments with three fish of each group per experiment. Scale bars = 10 µm. (C,D) Tg*(mpx:GFP)^i114^* fish with GFP-expressing neutrophils were used. (E–F) Tg(*mpeg1:GAL4/UAS:Kaede*) fish with Kaede-expressing macrophages were used. (C, E) Phagocytes were counted at the site of infection at 4 hpi. Total phagocytes includes all EGFP^+^ phagocytes (both with and without yeast) and EGFP^−^ phagocytes that internalized fungi. Phagocytes with *Candida* includes only phagocytes with internalized fungi. Data from at least three independent experiments was pooled and the average and standard error of all fish are shown. (D, F) At 4 hpi, fungi were scored as intracellular or extracellular, and the % internal was calculated per fish. Average and standard error are shown. *p<0.05 **p<0.01.

Given that NADPH oxidase is not required for intracellular containment of *C. albicans*, we sought another mechanistic explanation for its requirement in limiting filamentous growth. Our long-term timelapse experiments suggested that blockade of NADPH oxidase activity limited immune infiltration to the infection site (Fig. 1), and the Duox NADPH oxidase has been previously implicated in chemotaxis [19], [42]. To test if NADPH oxidase is required for early phagocyte chemotaxis to *C. albicans*, we treated fish with DPI and examined phagocyte dynamics in Tg*(mpx:GFP)^i114^* transgenic zebrafish [43], with EGFP-expressing neutrophils. Over the first four hours of infection, we found a reduction in the level of immune infiltration to the site of infection and an even stronger decrease in the amount of intracellular containment of fungi (Fig. 2A). Time-lapse imaging confirms that lower phagocyte numbers are due to loss of recruitment, rather than failure to retain phagocytes at the infection site. We quantified levels of infiltration and phagocytosis at 4 hours post-infection (4 hpi) as the total number of EGFP-positive cells (neutrophils) combined with the number of EGFP-negative cells (macrophages) with internalized *C. albicans* and found that total infiltration was significantly decreased (Fig. 2C). The number of total phagocytes with *C. albicans* inside was also significantly lower, as measured by the total number of immune cells, regardless of EGFP expression, that had engulfed *C. albicans* (Fig. 2C). Quantification of the number of internal vs. extracellular fungi showed that overall levels of engulfed fungi were significantly decreased by DPI treatment (Fig. 2D). Inclusion of both EGFP-positive neutrophils and EGFP-negative phagocytes—unambiguously scorable with internalized *C. albicans*—permitted a more robust measurement of the phagocyte response. Quantification of only the number of neutrophils at the infection site demonstrated a decreased number in DPI-inhibited fish, but the low overall number of EGFP-positive neutrophils at the site of infection led to differences that were not statistically significant (Fig. S5). In contrast to the effects on leukocyte infiltration, NADPH oxidase blockade did not strongly affect the overall ability of phagocytosis by neutrophils at the site of infection, as the percentage of neutrophils with engulfed fungi was largely unchanged, highly variable and not significantly affected by DPI treatment (Fig. S6). Taken together, these results indicate that short-term inactivation of NADPH oxidase activity evokes a significant deficiency in chemotaxis to the site of *C. albicans* infection.

Previous work has focused on NADPH oxidase-dependent neutrophil migration to the site of wounding, but macrophages also play an important role in response to *C. albicans* infection [31]. To test if NADPH oxidase activity is required for macrophage chemotaxis, we took advantage of the new Tg(*mpeg1:GAL4/UAS:Kaede*) line of zebrafish with macrophages expressing the photoswitchable Kaede fluorescent protein [38]. NADPH oxidase inhibition caused a significant decrease in macrophage migration to the infection site over the first 4 hpi, as shown by time-lapse microscopy (Fig. 2B). Quantifying these defects revealed significant reductions in total phagocyte infiltration and in total number of phagocytes with internalized fungi (Fig. 2E), similar to results using the neutrophil transgenic (Fig. 2C). In addition, we confirmed the strong defect in containment of fungi by phagocytosis using this transgenic with marked macrophages (Fig. 2F). Quantification of only the number of macrophages at the infection site demonstrated a decreased number in DPI-inhibited fish, a statistically significant difference (Fig. S5). The use of both neutrophil- and macrophage-specific transgenic lines allowed us to account for different types of phagocytes (either neutrophils or macrophages, depending on the transgenic line) that did not phagocytose fungi, as well as all phagocytes with intracellular fungi. Because the results of these two complementary sets of experiments are comparable, this implicates NADPH oxidase in chemoattraction of both phagocyte types to the site of infection. Our results suggest that NADPH oxidase-dependent leukocyte attraction then promotes phagocytosis primarily through efficient chemotaxis to the infection site rather than enhancement of engulfment at the infection site.

Serious tissue damage in the zebrafish larva elicits rapid neutrophil chemoattraction that is largely Duox-dependent [19], [42]. To test if tissue damage accounts for the NADPH oxidase-dependent attraction of neutrophils to the hindbrain ventricle upon *C. albicans* infection, we performed mock injections of buffer into vehicle (DMSO) or DPI-treated larva and measured neutrophil recruitment. We found that there was a small increase in hindbrain ventricle neutrophils in mock-injected larvae (from 0.75 to 2 neutrophils), although this was not significantly affected by DPI (2.05 vs 1.98) (Fig. S7). Thus, in contrast to the NADPH oxidase-dependent phagocyte recruitment to infection, the minor recruitment induced by this injection method is not NADPH oxidase-dependent.

Our data demonstrate that NADPH oxidase(s) direct the early immune response to fungal infection in the zebrafish hindbrain ventricle, tissue in the central nervous system. To test whether there is NADPH oxidase-dependent phagocyte recruitment and fungal containment in a localized infection in a different tissue, we infected the swimbladder of 4 dpf larvae with *C. albicans*. We have recently shown that the presence of *C. albicans* in the larval swimbladder elicits similar immune responses to those seen in an *in vitro* reconstituted human epithelial infection model [44]. Here, we modified the published protocol by injecting fungi directly into the swimbladder of 4 dpf larvae. We pre-incubated larvae with DMSO (vehicle) or DPI, injected 5–20 fungi/fish, maintained treatment for 4 hours, and then scored neutrophil migration to the infection site. We found that injection itself leads to a small but statistically significant increase in neutrophils at the site of infection (Fig. S8). This injection-associated increase is presumably due to a small amount of damage due to the injection procedure itself. Interestingly, this injection-related increase in neutrophil numbers is partially NADPH oxidase-dependent, as there is a small but significant reduction in neutrophil recruitment upon DPI treatment (Fig. S6). However, while injection of fungi led to a strong increase in neutrophil migration to the swimbladder, this increase was not DPI inhibitible. Thus, the early innate response to *C. albicans* infection in the swimbladder tissue at 4 dpf is different from the response in the hindbrain ventricle at 2 dpf, where in the swimbladder the early neutrophil response is more robust and not NADPH oxidase-dependent. These differences may be due to tissue-specific or stage-specific immune responses.

### The phagocyte NADPH oxidase is necessary for early phagocyte recruitment

**Figure 3:**
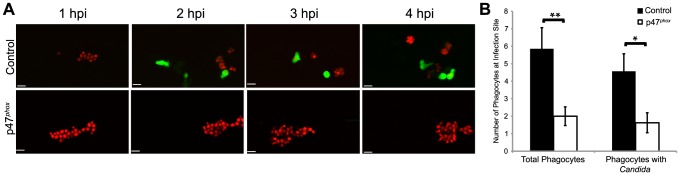
*Phox* morphants have impaired neutrophil migration. (A–B) CAF2-dTomato *C. albicans* were microinjected into the hindbrain ventricle of Tg(*mpx:GFP*)^i114^ control (n = 7) or p47*^phox^* morphants (n = 8) and imaged for 4 hours. (A) Representative time-lapse images of control and p47*^phox^* morphants. Results are representative of at least three experiments with at least two fish per group per experiment. Scale bars represent 10 µm. (B) Total phagocytes and phagocytes with internalized fungi were counted at 4 hpi. Numbers from fish over three experiments were pooled and mean and standard error per fish are shown. *p<0.05 **p<0.01 as calculated by two-tailed Student's T-test.

We have shown that efficient engulfment of *C. albicans* in the hindbrain ventricle depends on NADPH oxidase-mediated phagocyte recruitment, and that internalization blocks switching from yeast to filamentous form. We therefore speculated that the increased filamentous growth previously observed upon knockdown of p47*^phox^* [31] was due to defective early chemotaxis and containment. We tested the requirement of p47*^phox^* for early immune response by measuring immune responses to infection in p47*^phox^* morphants, with reduced phagocyte oxidase activity. Using the Tg*(mpx:GFP)^i114^* line, we find that p47*^phox^* knockdown leads to chemotaxis deficits similar to that of DPI treatment. This is seen both with time-lapse imaging (Fig. 3A) and upon quantitation of phagocyte behavioral phenotypes at 4 hpi (Fig. 3B). The similar immune deficits upon pan-NADPH oxidase inhibition and p47*^phox^* knockdown suggest that the phagocyte oxidase Phox is an important mediator of phagocyte migration and intracellular containment of *C. albicans*. Further, this suggests that the previously described increase in filamentous growth seen at 24 hpi in p47*^phox^* morphants [31] is a direct consequence of early defects in fungal containment.

### The epithelial Duox NADPH oxidase is also required for early phagocyte chemotaxis

**Figure 4:**
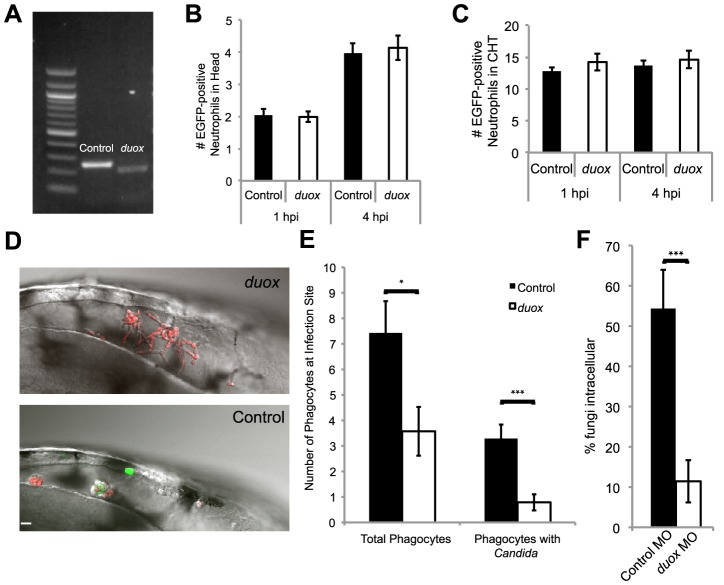
*Duox* knockdown phenocopies p47*^phox^* knockdown and DPI treatment. (A–F) Control and *Duox* morpholinos were co-injected with *p53* morpholino into 1-cell stage Tg(*mpx:GFP*)^i114^ zebrafish embryos to create morphants. (A) Samples were collected and prepared for RT-PCR verification of morpholino knockdown. A 39 base pair deletion in the *duox* message is observed in morphants. (B) Basal level of neutrophils in head at time of infection. Control and *duox* morphant Tg(*mpx:GFP*)^i114^ fish were injected at the prim25 stage with PBS to simulate infection. At 1 hpi and 4 hpi neutrophils in the head were counted at 1 hpi; n = 68 controls and 77 *duox* morphants, at 4 hpi n = 70 controls and 71 *duox* morphants. Data pooled from 5 independent experiments. (C) Basal levels of neutrophils in caudal hematopoetic tissue (CHT). Control and *duox* morphant Tg(*mpx:GFP*)^i114^ fish were PBS-injected at the prim25 stage and imaged at 1 hpi and 4 hpi. Data shown are representative of three independent experiments; n = 16 control and n = 13 *duox* morphants. (D–F) Phagocyte migration. Control (n = 15) and *duox* (n = 15) morphant Tg(*mpx:GFP*)^i114^ fish were injected at the prim25 stage with CAF2-dTomato and imaged until 4 hpi. (D) Representative images of infection site show severe reduction in phagocytosis and extensive extracellular filamentous growth in *duox* morphants (top) compared with controls (bottom). Scale bar = 10 µm. Representative of three independent experiments. (E) Phagocytes were counted at the site of infection at 4 hpi. Total phagocytes includes all EGFP+ neutrophils and EGFP- phagocytes that internalized fungi. Phagocytes with *Candida* includes only phagocytes with internalized fungi. Data from all experiments was pooled and the average and standard error of all fish are shown. (F) At 4 hpi, fungi were scored as intracellular or extracellular, and the percent internal was calculated per fish. Data from three independent experiments were pooled and the average per fish and standard error are shown. *p<0.05 **p<0.01 ***p<0.001.

Our results indicate that the phagocyte oxidase is required for phagocyte chemotaxis, suggesting a requirement for NADPH oxidase activity in leukocytes. Although not previously implicated in chemotaxis to microbes, the epithelial NADPH oxidase Duox is highly expressed in the brain and has a previously defined role in neutrophil chemotaxis to wounds [19], [42], [43], [45]. To test a role for Duox in phagocyte chemotaxis to *C. albicans*, we knocked down expression of *duox*, confirmed knockdown by rtPCR as described [42], and verified that this morpholino eliminated all detectable transcript without causing gross developmental effects (Fig. 4A). To assess global and local neutrophil numbers within prim25 zebrafish, we counted EGFP-positive neutrophils in the head and caudal hematopoetic regions of *duox* and control morphants in a mock experiment, both 1 hour and 4 hours after injection with phosphate-buffered saline. We found no difference in basal numbers of EGFP-positive neutrophils in either tissue of *duox* morphants at the stages of development most relevant to our studies (Fig. 4B and 4C). In contrast to our expectations, immune responses to infection in the *duox* morphants were severely impaired, similar to what we find with chemical inhibition and with knockdown of p47*^phox^*. This is seen with time-lapse imaging (Fig. 4D), quantitation of phagocyte behavioral phenotypes at 4 hpi (Fig. 4E), and overall failure of phagocytosis (Fig. 4F). Interestingly, we do find a trend toward decreased phagocytosis on a per-cell basis for *duox* morphant neutrophils that is more consistent than trends seen for both DPI-treated and p47*^phox^* morphant neutrophils (Fig. S2). This stronger phenotype suggests the possibility that Duox plays a more important role than p47*^phox^* in directing the phagocytic process, although the small number of neutrophils at the infection site in these treated fish makes such characterizations necessarily tentative. In sum, the phenocopy of DPI treatment in both p47*^phox^* and *duox* morphants suggests that both the phagocyte-expressed Phox and the non-phagocyte-expressed Duox are required for bringing phagocytes to the site of infection, thus promoting efficient phagocytosis and inhibition of germination.

### Reduced phagocytosis leads to extracellular filamentous growth and mortality

**Figure 5:**
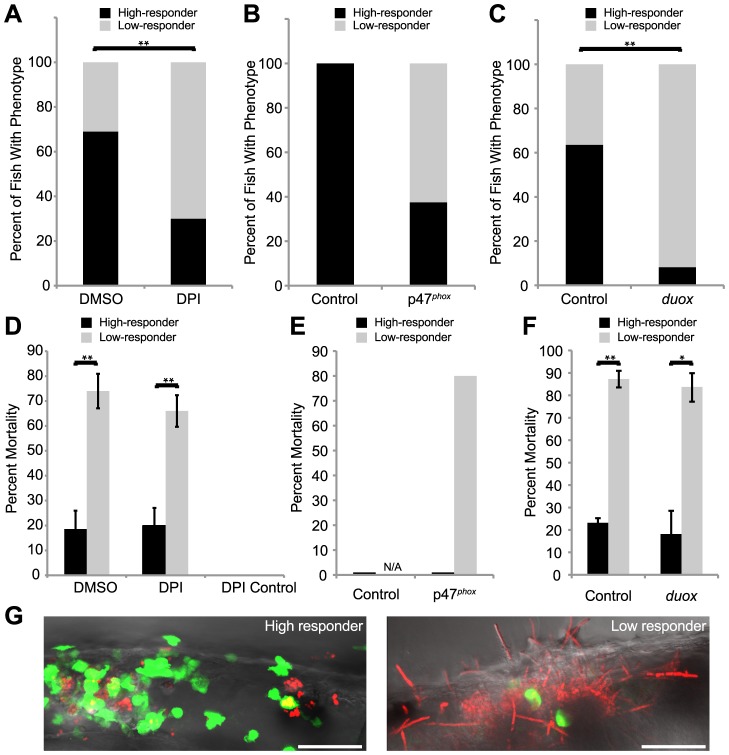
Weak early immune response permits filamentous growth and promotes pathogenesis. (A–F) Tg(*mpx:GFP*)^i114^ fish with green fluorescent neutrophils were infected with CAF2-dTomato at the prim25 stage, imaged at 4 hpi by microscopy to quantify phagocytosis efficiency, then sorted into individual wells in a 24-well dish and scored for survival at 24 hpi. (A–C) Low and high responders were quantified at 4 hpi by microscopy. Low responders had five or greater extracellular fungi at 4 hpi. (A,C) Data shown are means from three independent experiments that were analyzed by two-tailed Student's T-test. (B, E) Data shown are pooled from three independent experiments, as the number of total fish was too small (n = 6) for similar statistical analysis. (D–F) The percent of high or low responders that died by 24 hpi was quantified. Data shown are either the averages and standard errors (D and F) or were pooled (E) from three independent experiments. Statistical analysis was performed by Student's T-test (D and F). (A, D) Infected fish were treated with DMSO (n = 131) or DPI (n = 118) from 2 hours pre-infection to 4 hpi. (B, E) Control (n = 7) or p47*^phox^* (n = 8) morphants were infected and followed. (C,F) Control (n = 129) or *duox* (n = 139) morphants were infected and followed. (G) High responders (left) contain the infection by 24 hpi, in contrast to low responders (right) which permit filamentous growth and more frequently die by 24 hpi. Images are representative of three independent experiments. Scale bar = 50 µm. N/A = not applicable because no fish in this category. *p<0.05, **p<0.01.

Our observations demonstrate that NADPH oxidases act quickly post-infection to attract phagocytes to *C. albicans* and limit its filamentous growth by internalization. To understand the importance of these early immune responses, we sought to identify the consequences of poor initial infiltration and phagocytosis. Our previous work established that failure to control filamentous growth at 24 hpi correlates with poor survival to 48 hpi [31], and here we again exploited non-invasive imaging to ask if weak early phagocytosis is linked to extracellular filamentation and poor prognosis. We characterized infected fish at 4 hpi as “low” or “high” responders, depending on whether they had greater or fewer than five extracellular fungi. Surprisingly, there were fish of each type for each treatment group, including the control groups, indicating that there is some heterogeneity in immune competence among genetically identical individuals infected with the same doses. However, consistent with our time-lapse results, there were more low responders in DPI-treated (Fig. 5A), p47*^phox^* morphants (Fig. 5B) and *duox* morphants (Fig. 5C) than the comparable controls. Thus, despite screening individual fish immediately post-infection to ensure consistent infectious doses, by 4 hpi the infections could be classified into two major categories dependent on the efficiency of intracellular containment. As expected, the number of high-responder fish was strongly decreased by all treatments that blocked NADPH oxidase activity.

To determine if these early phenotypes are prognostic for survival, we assayed the fate of individual fish screened at 4 hpi. Fish were imaged and scored for phagocyte response at 4 hpi, then kept in individual wells of a 24-well plate until 24 hpi to assess their fate. As expected, low responders have a much worse prognosis than high responders, with approximately three-quarters succumbing to infection by 24 hpi (Fig. 5D–5F). Remarkably, though, the prognosis among low responders is comparable between controls and treatment groups, and the same is true for high responders. Due to the role of phagocytosis in limiting germination, low responders have excessive filamentous fungal growth, and nearly all of the fish that die by 24 hpi are riddled with *C. albicans* filaments (Fig. 5G). The close correspondence of early phagocytosis with infection containment and survival highlights the crucial importance of early NADPH oxidase activity in protecting the host against *C. albicans*. Considering the similar phenotypes between temporary chemical blockade and long-lasting morpholino knockdown, this suggests that early NADPH oxidase activity plays a more important role than later production of ROS in control of this acute disease.

### The *EDT1* dimorphic switching program limits early immune chemotaxis and overall virulence

**Figure 6:**
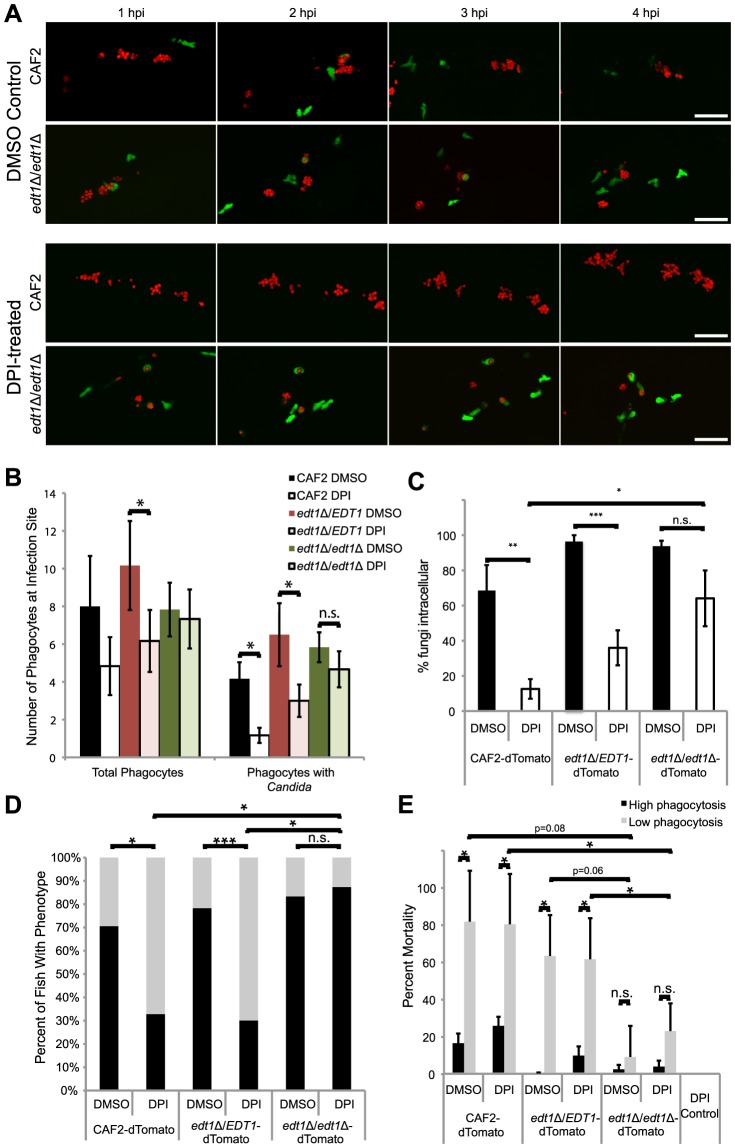
The *EDT1*-dependent morphogenetic switching pathway plays roles in NADPH oxidase-independent phagocyte migration and virulence. (A–E) prim25 stage Tg(*mpx:GFP*)*^i114^* larvae were injected with CAF2-dTomato, *edt1*Δ/Δ-dTomato, or *edt1*Δ/*EDT1*-dTomato *C. albicans*. Fish were incubated with DMSO or DPI from two hours pre-infection until 4 hpi, and imaged by confocal microscopy. At 4 hpi phagocyte migration and phagocytosis were quantified and fish were sorted into high- and low-responder categories, and at 24 hpi fish were scored for survival. (A) Images are representative of three independent experiments; n = 6 for each fungal genotype and treatment group. Time-lapse images from *edt1*Δ/*EDT1* infections are indistinguishable from wildtype infections but are not shown due to space considerations. Scale bar = 50 µm. (B) Quantitation of total phagocyte response and number of phagocytes with internalized fungi shows the average and standard error from fish pooled from three independent experiments; n = 6 for each fungal genotype and treatment group. (C) Phagocytosis efficiency was measured in fish pooled from three independent experiments; n = 6 for each fungal genotype and treatment group. (D) Survival percentage of low- and high-responders was measured for each of three independent experiments, and the means are shown; n = 70–74 for each group. (E) Low mortality of low-responders infected with yeast-locked *edt1*Δ/Δ mutant. Means and standard errors of mortality percentages of low- and high-responders at 24 hpi are shown. (B–E) Data represent three independent experiments. Means and standard errors are shown. Differences between groups were assessed by two-tailed Student's T-test (B, C, and E) or Fisher's exact test (D). *p<0.05, **p<0.01, ***p<0.001, n.s. no significant difference.

Our demonstration of NADPH oxidase-dependent phagocyte recruitment is in contrast to what has been seen with other pathogens [15], [18], suggesting that *C. albicans* may have a special ability to counter ROS-independent chemotaxis. Because the yeast-to-hyphal switch is an important virulence trait associated with genome-wide transcriptional remodeling [46], [47], we hypothesized that it may be required to limit NADPH oxidase-independent chemotaxis. To test this idea, we examined the effects of NADPH oxidase inhibition on infections with the yeast-locked *edt1*Δ/Δ mutant. We infected Tg*(mpx:GFP)^i114^* fish with three different strains of dTomato-expressing *C. albicans*: wildtype, homozygous *edt1*Δ/Δ mutant, or heterozygous *edt1*Δ/*EDT1* control. We used the heterozygous *edt1*Δ/*EDT1* mutant to control for potential artifacts due to transformation. We treated infected fish with DPI or vehicle and performed time-lapse experiments to measure early immune response. To our surprise, we found that a high proportion of DPI-treated, *edt1*Δ/Δ-infected fish elaborate a strong early immune response in which most of the fungi is internalized (Fig. 6A). Infections with the heterozygous *edt1*Δ/Δ/*EDT1* control result in an intermediate phenotype, as is found frequently with mutants in the diploid *C. albicans*. Quantitation of this response in even the limited number of fish examined by time-lapse microscopy suggests that there is a similar level of overall immune recruitment to the *edt1*Δ/Δ infection site, independent of NADPH oxidase inhibition (Fig. 6B). Internalization of *edt1*Δ/Δ is also apparently NADPH oxidase-independent, and a much higher percentage of yeast-locked fungi than wild type fungi are phagocytosed by 4 hpi (Fig. 6C). Percent phagocytosis is intermediate for the heterozygous *edt1*Δ/Δ/*EDT1* strain, suggesting that there may be a partial haploinsufficiency phenotype. Consistent with these high-resolution time-lapse results with a small sample size, we also find a large percentage of high responders in *edt1*Δ/Δ-infected, DPI-treated fish when large numbers of fish are screened at 4 hpi for their ability to contain the fungi (Fig. 6D). The significant difference in NADPH oxidase-independent phagocyte migration to the yeast-locked mutant in fungal containment at 4 hpi suggests that changes in *C. albicans* during the dimorphic switch may play an important role in limiting phagocyte chemotaxis.

Our data demonstrate that germination of extracellular *C. albicans* is enhanced by poor early immune response, which is associated with poor prognosis. We therefore reasoned that genetic blockade of the *C. albicans* dimorphic switch would prevent mortality, even in conditions of poor phagocyte containment. To investigate the contribution of the dimorphic switching program to virulence under these circumstances, we followed the fate of high and low responder fish infected with the yeast-locked *edt1*Δ/Δ mutant. Although the majority of *edt1*Δ/Δ-infected fish internalize fungi successfully, the naturally heterogeneous early immune response among individuals allowed testing of our original hypothesis that extracellular germination is responsible for the poor prognosis after weak early chemotaxis. As expected, the outcome of infections in low-responding fish diverges significantly between *edt1*Δ/Δ- and control-infected fish. In contrast to the situation with control-infected fish, most of the low responders infected with *edt1*Δ/Δ manage to survive to 24 hpi and beyond (Fig. 6E). As is the case for other infections, there are no NADPH oxidase-dependent differences in mortality within high- and low-responder groups. Thus, even when the early immune response fails to successfully contain the majority of *edt1*Δ/Δ mutant fungi, their inability to turn on the dimorphic switching pathway prevents pathogenesis. These data point to the importance of the *EDT1*-dependent dimorphic switching pathway in both limiting early fungal containment and in exploiting a weak early response to grow extracellularly in filamentous form and cause mortality.

## Discussion

The advent of intravital imaging has begun to illuminate new aspects of host-pathogen interaction in the intact host. Here, we exploited a transparent zebrafish model of candidemia to address mechanistic questions relevant to human primary immunodeficiency and immune response dynamics. We describe a new role for NADPH oxidase in recruitment of phagocytes to the site of *C. albicans* infection, demonstrate that this early recruitment is a key event in control of infection, and provide evidence that the *C. albicans* dimorphic growth program impacts the ROS-dependence of early fungal containment. The discovery of this unanticipated role of NADPH oxidase in phagocyte recruitment highlights the importance of early immune responses and points to a potentially new role of fungal dimorphism in regulating phagocyte activity.

In this study, we used a powerful *in vivo* model to demonstrate a role for NADPH oxidase-driven phagocyte containment of *C. albicans*. Classically, ROS produced by the phagocyte oxidase and the dual-specific oxidase have been ascribed functions in direct chemical attack against systemic and epithelial insults [9], [48]. Although we find no evidence for a role of ROS in directly damaging intracellular *C. albicans* early during infection *in vivo*, we ascribe a novel role to these two NADPH oxidases in recruitment of leukocytes to the site of *C. albicans* infection. In addition to our findings, abundant recent work challenges the narrow view of ROS as solely microbicidal and implicates NADPH oxidase-produced ROS in a range of other functions such as autophagy, neutrophil extracellular traps, tryptophan metabolism, kinase signaling, neutrophil recruitment to the endothelium and epithelium, and inflammasome activation [9], [19], [35], [48]–[56]. In this context, it is notable that we did not find frequent LC3-associated phagocytosis (LAP) of fungi, in contrast to what has been observed *in vitro* [35]. Perhaps most relevant to our findings is recent work suggesting a role for the phagocyte NADPH oxidase or Duox in enabling neutrophil chemotaxis *in vivo* [16], [19], [42], [52]. Our work highlights how this latter function of NADPH oxidases in chemoattraction can play an important role in immunity to the fungal pathogen *C. albicans* in this zebrafish model.

The implication of both Phox and Duox in driving early immune recruitment was unexpected, despite their shared capacity to produce hydrogen peroxide. The consequences of pan-NADPH oxidase inhibition by DPI are no more dramatic than inhibition of either Phox or Duox, suggesting that inhibition of both enzymes is comparable to inhibition of either one alone. The largely non-redundant phenotypes of p47*^phox^* and Duox knockdowns imply that these two enzymes may work together in the same pathway or complex, but it is unlikely that p47*^phox^* directly control Duox, as Duox regulation is only known to occur through Ca^2+^ [57]–[59]. Alternatively, they may play separate, indispensable roles in the same or different tissues. We do not know if the relevant activity of these NADPH oxidases is within leukocytes, epithelial cells, or both cell types. Recent work identified a novel function for Phox within human and mouse neutrophils in chemotaxis toward defined chemoattractants *in vivo* and *in vitro* and toward sterile inflammation *in vivo* [16]. While Phox is expressed most highly in phagocytes, it is expressed widely and has recently been shown to have important activities in other tissues [57], [60]. On the other hand, Duox in epithelial cells has an important function in signaling infection and attracting leukocytes to damage [48], [52]. In zebrafish, Duox has been shown to play a role in neutrophil chemotaxis to wounds, but may not be involved in leukocyte chemotaxis to bacterial infections [15]. Tissue-specific promoters in zebrafish should enable determination of the cell types requiring NADPH oxidase components in fungal infection.

If phagocyte oxidase is not required to limit intracellular germination, what mechanisms contain internalized *C. albicans*? Together with previous work [31], our data suggest that NADPH oxidases play an early role in phagocyte recruitment but not in prevention of germination. This suggests that early during infection other phagocyte mechanisms ensure containment and prevention of *C. albicans* filamentous growth within phagocytes. While phagocyte oxidase is required for the PMA-stimulated respiratory burst, our work here shows this role does not seem to impact early attack on *C. albicans*, as measured by oxidative stress or phagocyte activation. Consistent with the selective role of Phox-mediated oxidative damage *in vivo*, only a subset of all *C. albicans* experiences oxidative stress at a given time during systemic infection in the mouse [32]. Instead, phagocytes have been shown to have the capacity to use NADPH oxidase-independent mechanisms to limit *C. albicans* growth, limit germination, and in some cases kill intracellular fungi. Notably, NOX2 −/− NOS2 −/− mice missing essential components of the phagocyte oxidase and inducible nitric oxide synthase are still able to limit *C. albicans* virulence *in vivo* in a gut infection model and macrophages from these mice can kill *C. albicans in vitro* [22]. Several lines of evidence also suggest neutrophil proteases are important in damaging *C. albicans* and limiting its virulence [23], [25]. Furthermore, *C. albicans* is sensitive to antimicrobial peptides [61] and neutrophil extracellular traps [26]. New technical advances may enable the determination of the dynamics of fungal killing by these mechanisms in mice, which have never been directly assayed through the type of continuous observation shown here in the zebrafish.

What are the consequences of a poor initial immune response to *C. albicans* infection? In both normal and NADPH oxidase-inhibited fish, failure to contain *C. albicans* in the first four hours is tantamount to overall failure to eliminate the pathogen. *C. albicans* is prevented from germination within neutrophils and macrophages for at least the first 24 hpi. In contrast, extracellular *C. albicans* germinates into tissue, and the fungus' ability to turn on the *EDT1* transcription factor allows both dimorphic switching and prevention of NADPH oxidase-independent containment. Both of these activities play important roles in virulence. Switching to hyphal form inhibits phagocytosis [62] and permits tissue invasion [63], leading to florid filamentous growth that destroys host tissue. Effective immune migration to the infection site is a strong prognostic indicator of survival, regardless of the presence of functional NADPH oxidases. The early role of NADPH oxidases is also emphasized by the effect of a temporary, 4 hour, blockade through DPI incubation. In this case, despite wash-out of the drug afterwards, phagocytosis efficiency at 4 hpi predicted survival. Thus, later activity of NADPH oxidase, which mediates fungal oxidative stress, is still not effective at stopping the infection. The importance of early immune response is clear in the mouse, as well, where delayed immune infiltration into the kidney plays an important role in making this organ much more susceptible to *C. albicans* proliferation [64], whereas late-infiltrating neutrophils [65] cause excessive damage, a scenario that is more than just a case of “too-little, too-late”.

If NADPH oxidases are not required for phagocyte chemotaxis to bacterial infections in the zebrafish [15], [18], why are they required for recruitment to *C. albicans*? Response to these other infections likely involves redundant immune mechanisms including ROS and other chemotactic cues for ensuring leukocyte chemotaxis. The finding that fungal containment of the yeast-locked *edt1*Δ/Δ mutant is not significantly reduced by NADPH oxidase-inhibition suggests that specific pathogen mechanisms may limit the immune response and force a dependence on ROS. Recent *in vitro* work shows that *C. albicans* hyphae are harder to phagocytose than yeast, which suggests that fungi that escape phagocytosis and germinate extracellularly early during infection in the zebrafish will be able to resist phagocytosis later [62]. Accordingly, this may impact the phagocytosis of yeast-locked *edt1*Δ/Δ mutants. The potential link between dimorphic switching and blockade of ROS-independent phagocyte chemotaxis suggests yet another way that the ability to switch to an invasive hyphal form may contribute to *C. albicans* virulence *in vivo*, as has been shown in both mucosal and disseminated candidiasis in the mouse [66], [67]. Additional virulence mechanisms depend on the germination program, because even poor early phagocytosis of *edt1*Δ/Δ mutant fungi did not always lead to mortality. There are several known mechanisms whereby *C. albicans* regulates interaction with host leukocytes. Live *C. albicans* blocks phagocyte ROS production [68], [69], and hyphally derived soluble products block PMN activation [70]. There are a number of transcriptional pathways co-regulated with the *EDT1*-mediated yeast-to-hyphal transition, and these may contribute to the differential virulence of the mutant. The transparent model exploited here may prove useful in dissecting how host and fungal determinants govern leukocyte chemotaxis early during infection.

While our work using the hindbrain ventricle model of infection is the first to show this new role of NADPH oxidases in early fungal containment, dependence on this mechanism may be pathogen-, developmental- or tissue-specific. Our findings that neutrophil migration to the swimbladder in 4 dpf old zebrafish is NADPH oxidase-independent, in contrast to the NADPH oxidase-dependence in the 2 dpf old hindbrain ventricle model suggests that different mechanisms may be at play in different tissues and/or at different times of development. Thus, the central nervous system may rely more heavily on ROS to drive phagocyte recruitment than other tissues such as the swimbladder mucosa, which shares common ontogeny and gene expression with the mammalian lung [71], [72]. The tissue-specificity we observe is mirrored in recent work with a different fungal pathogen, *Cryptococcus neoformans*, which suggests that immune cell recruitment mechanisms are different in the brain and lung [73]. In addition, the lack of NADPH oxidase-dependence in neutrophil responses to bacterial infection of the otic vesicle at 3 dpf suggests that there may be developmental- and/or microbe-specific roles of these enzymes in early immune response [15]. A negative role for NADPH oxidase in neutrophil chemoattraction has been suggested by several mouse models of sterile inflammation as well as in chronic granulomatous disease [9], although its potential early role in response to fungi remains to be tested. There are likely to be multiple roles of NADPH oxidases in immunity, but our non-invasive time-lapse experiments highlight a previously unappreciated early role for Phox and Duox in leukocyte activity that recent work suggests may be evolutionarily conserved in mammals [16].

Non-invasive imaging of spatiotemporal immune responses with photoswitched Kaede-expressing macrophages revealed a reduced mobility of macrophages with internalized fungi. This could reflect different types of macrophages at the site of infection, similar to the M1 and M2 macrophages in mammals that express different sets of immune receptors and therefore have differential ability to uptake microbes [40], [74]–[77]. M1 and M2 macrophages also differentially express cytoskeletal modulators such as cadherins [78]. Alternatively, the relatively low mobility of macrophages with internalized fungi could result from macrophage differentiation upon activation in response to pathogen recognition. Differentiation-induced loss of motility is a well-characterized developmental paradigm [79] well described for border cells in *Drosophila melanogaster* [80] and neural crest in zebrafish [81]. Consistent with this idea, phagocytes have been shown to undergo significant cellular rewiring upon pathogen recognition *in vitro* [82], and activation of receptors alters the cytoskeleton and thereby macrophage motility *in vivo* and *in vitro* [83], [84]. Regulation of phagocyte motility in response to pathogen recognition is still poorly understood *in vivo*, yet may play important roles in pathogen containment and fine-tuning of the host response.

**Figure 7:**
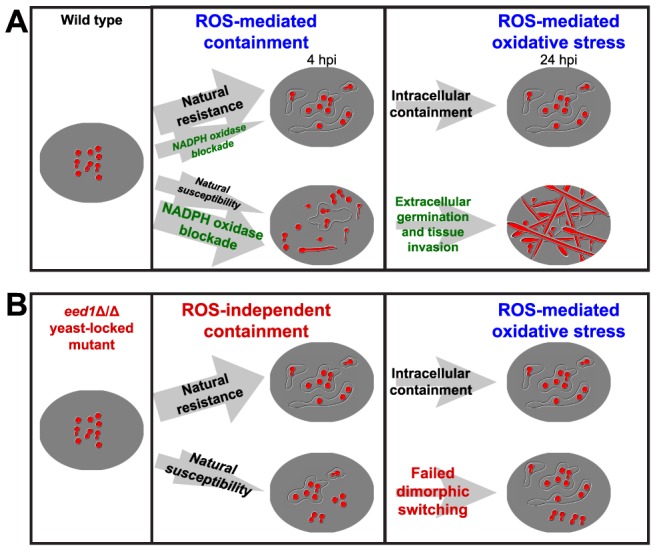
A revised model of NADPH oxidase activity in the context of *C. albicans* infection. (A) Naturally, the majority of larvae mobilize phagocytes rapidly to the site of infection and phagocytose nearly all fungi by 4 hpi, as represented by the upper pathway. A minority of larvae fail to mobilize enough phagocytes to internalize most fungi by 4 hpi, as represented by the lower pathway. NADPH oxidase activity blockade by chemical treatment or gene knockdown (green) shifts this balance towards the lower pathway, in which germination of *C. albicans* extracellularly leads to tissue invasion and death by 24 hpi. NADPH oxidase production of ROS (blue) is required early for phagocyte recruitment and late for attacking fungi and causing oxidative stress. (B) Phagocyte mobilization in response to infection with the yeast-locked *edt1*Δ/Δ mutant does not depend on NADPH oxidase-produced ROS (blue). In the mutant infections there may be additional chemoattractants produced and/or a failure to block ROS-independent recruitment. This mutant is yeast-locked (red), and therefore does not germinate extracellularly, invade or cause high levels of mortality.

As illustrated in our model (Fig. 7), these results suggest several new facets of the role of NADPH oxidase in early events of the hindbrain ventricle *C. albicans* infection. First, ROS produced by Phox and Duox are required for recruitment of phagocytes to the infection site. Second, the continued recruitment of phagocytes to the infection site requires NADPH oxidase activity. Third, attraction of phagocytes to the yeast-locked *edt1Δ*/Δ mutant does not require ROS and may be mediated by fungal products and/or host chemoattractants that are produced in an ROS-independent fashion. Fourth, effective recruitment of phagocytes leads to efficient phagocytosis, containment of fungi, and survival of the challenge. This dynamic picture challenges the existing paradigm for NADPH oxidase-mediated immune responses and highlights a need for further research into host and fungal regulation of chemotaxis and containment.

## Materials & Methods

### Zebrafish care and maintenance

Zebrafish were kept in recirculating systems (Aquatic Habitats, Apopka, FL) at the University of Maine Zebrafish Facility. All zebrafish care and husbandry were performed as described previously [85]. All zebrafish care protocols and experiments were performed in accordance with NIH guidelines under Institutional Animal Care and Use Committee (IACUC) protocol A2009-11-01. Larvae were grown at a density of 50/dish in 10-cm Petri dishes containing 60 ml of egg water (deionized water with 60 mg/L Instant Ocean salts [Spectrum Brands, Mentor, OH]). The fish strains used were wild-type AB and the transgenic strains Tg(*mpx:GFP*)*^i114^* [43] expressing enhanced green fluorescent protein in neutrophils, Tg(*mpeg1:GAL4/UAS:Kaede*) expressing the photoswitchable fluorescent protein Kaede in macrophages [38], and Tg(*GFP:Lc3*) expressing enhanced green fluorescent protein fused to LC3 [37]. The Tg(*mpeg1:GAL4/UAS:Kaede*) line has been extensively characterized and shown to provide macrophage-specific gene expression at this developmental stage [38]. Given the infection site in the hindbrain, it is also important to note that differentiation of microglia does not begin until 70 hpf, nearly 36 hours after the prim25 stage used in this infection model [86]. Egg water was supplemented with 0.3 mg/L methylene blue for the first 24 hours to prevent microbial growth. Larvae were cleaned by changing the egg water daily. For Tg(*mpeg1:GAL4/UAS:Kaede*) overnight imaging experiments, zebrafish were raised in water containing 15 µg/mL of 1-phenyl-2-thiourea (PTU) (Sigma-Aldrich, St. Louis, MO) to prevent pigmentation.

### Fungal strains and growth conditions

*C. albicans* strains were grown on yeast-peptone-dextrose (YPD) agar (Difco; 20 g/L peptone, 10 g/L yeast extract, 20 g/L glucose, 2% agar). For infections, liquid cultures of *C. albicans* were grown overnight with shaking in YPD broth (DIFCO; 20 g/L peptone, 10 g/L yeast extract, and 20 g/L glucose) at 37°C. Overnight cultures were washed three times in calcium-and magnesium-free phosphate-buffered saline (PBS; Lonza, Walkersville, MD), counted on a hemocytometer, and adjusted to a concentration of 1×10^7^ cells/ml. OxYellow-T, CAF2-dTomato, *edt1*Δ/Δ-dTomato and *edt1*Δ/*EDT1*-dTomato strains were created by transforming the original CTA1-GFP [32], CAF2 [87], *edt1*Δ/Δ and *edt1*Δ/*EDT1* [88], [89] strains with a construct containing the P*_ENO1_* promoter, a codon-optimized dTomato gene, the T*_TEF_* terminator, and a NAT^r^ marker. This dTomato construct is described in more detail in Gratacap et al. [44]. Transformants were selected on 100 µg/ml nourseothricin, and screened both for correct integration at the *ENO1* locus by PCR and for bright fluorescence by flow cytometry.

### Morpholino knockdown

Modified antisense oligonucleotides (MOs) designed to knock down translation of p47*^phox^* (NCF1: 5′-CGGCGAGATGAAGTGTGTGAGCGAG, [31]), or to block splicing of *duox* (5′-AGTGAATTAGAGAAATGCACCTTTT, [42]) were synthesized by Gene Tools (Philomath, OR). Morpholinos were reconstituted in nuclease-free water, and appropriate dilutions were stored in Danieau buffer (58 mM NaCl, 0.7 mM KCl, 0.4 mM MgSO_4_, 0.6 mM Ca(NO_3_)_2_, 5.0 mM HEPES, pH 7.6). A standard control morpholino from Gene Tools was used at the indicated doses in all experiments. Morpholinos were injected into 1-cell embryos in 5 nl volumes to achieve a final dose of 2.5 ng of p47*^phox^*, and 100 µM (4.25 ng) *duox*. Stocks were prepared in 0.01% phenol red for visualization of injection success. For *duox* MO experiments, embryos were co-injected with 300 µM (11.7 ng) *p53* morpholino, 5′-GCGCCATTGCTTTGCAAGAATTG, as previously described [42].

### Hindbrain ventricle infection

Infections were carried out as described [31]. Zebrafish at the prim25 stage (approximately 36 hours post fertilization) were staged according to the method of Kimmel, *et al.* [90], manually dechorionated, and anesthetized in Tris-buffered tricaine methane sulfonate (tricaine; 200 µg/ml) (Western Chemicals, Inc., Frendale, WA). For infection, 5 to 10 nL of PBS or *C. albicans* suspension at 1×10^7^cells/mL in PBS was microinjected through the otic vesicle into the hindbrain ventricle to achieve a dose of approximately 10 yeast/larva. Fish were screened by microscopy immediately post-infection to ensure correct inoculum sizes and injection location.

### Swimbladder injection

Two hundred *mpx:GFP* zebrafish were collected and incubated overnight in E3 media plus methylene blue at 33°C in 15-cm Petri dishes. Fish were kept at a density of 120 fish per dish with 150 mL E3. At 1 dpf, the media was changed to E3+PTU (20 µg/mL final concentration) and the fish were incubated for 3 days at 33°C. At 4 dpf, *mpx:GFP* fish were screened for overall number and distribution of neutrophils; only fish with a homogenous and representative number of neutrophils, as well as an inflated swimbladder were selected. Two groups of 90 fish were treated with vehicle (0.8% DMSO) or DPI (100 µM) for one hour in the dark. All fish were anaesthetized in tricaine (as previously described). Thirty (30) fish from each group were left un-injected, 30 fish from each group were injected in the swimbladder with 3 nL of PBS and the remaining 30 fish from each group were injected in the swimbladder with 3 nL of 1.5×10^7^ CFU/mL of *C. albicans* CAF2-dTomato *C. albicans*. The latter group was immediately screened by epifluorescence in 96-well-plate imaging dishes (Greiner BioOne SensoPlates). Fish with an inoculum between 5 and 20 yeast cells in the swimbladder were selected and incubated for 4 hours in E3+vehicle (DMSO) or DPI in the dark. Fish were then imaged by confocal microscopy in 0.4% low melt agarose+tricaine+vehicle (DMSO) or DPI. The number of neutrophils at the site of infection was recorded and 30 z-slices were acquired through the swimbladder (approximately 100–150 µm depth) in the green (488/510 nm), red (547/618 nm) and differential interference contrast (DIC) channels from representative fish. Images are composites of maximum projections for the red and green channels (number of slices indicated in the figure legends) and of a single middle slice for the DIC channel.

### Fluorescence microscopy

An Olympus IX-81 inverted microscope with an FV-1000 laser scanning confocal system was used for confocal imaging (Olympus). Objective lenses with powers of 4×/0.16 NA, 10×/0.4 NA, 20×/0.7 NA, and 40×/0.75 NA were used. Larvae were anesthetized in Tris-buffered tricaine methane sulfonate (200 µg/ml) and further immobilized in a mixture of 0.5% low-melting point agarose (Lonza, Walkersville MD) in egg water including the same amount of tricaine. Images are overlays of fluorescence image panels (red-green) or overlays of differential interference contrast (DIC) and fluorescence images. dTomato, EGFP, unconverted and photoswitched Kaede were detected by optical filters for excitation/emission at 543 nm/610 nm, 488 nm/510 nm, 460–480 nm/495–540 nm, and 540–580 nm/610 nm, respectively. To photoswitch Kaede locally, larvae were imaged at 40×/0.75 NA with only the hindbrain ventricle being exposed to laser light. Larvae were then subjected to 15 minute exposure of violet light by scanning of the 405 nm laser at 5% power using the Fluoview X-Y repeat setting. Images post-photoswitching were captured by 10×/0.4 NA, 20×/0.7 NA, and 40×/0.75 NA to confirm only macrophages in the hindbrain ventricle had been photoswitched from green to red fluorescence. Mortality of infected photoswitched larvae (37.5%) compared well to mortality of infected non-photoswitched larvae (30.3%), suggesting that photoswitching does not cause significant damage to the zebrafish (Fig. S4). For all time courses larvae were kept in low-melting-point agarose with egg water and tricaine in cover glass-bottom 24-well dishes (MatTek, Ashland MA), kept at a constant temperature of 28°C, and imaged over time with an Olympus-FV-1000 laser scanning confocal system. Z-stacks from time course images were manually analyzed and the number of *C. albicans* and fluorescent phagocytes were quantified.

### H_2_DCF-DA imaging

Six groups of thirty AB prim25 fish were anaesthetized with tricaine as previously described. Two groups of thirty fish were left uninjected, two groups of thirty fish were injected in the hindbrain with 3 nL of PBS and two groups of thirty fish were injected in the hindbrain with 3 nL of 1.5×10^7^ cfu/mL of CAF2-dTomato *C. albicans*. One group for each treatment was incubated for 1 hour in a solution of E3+in a final concentration of 500 ng/mL H_2_DCF-DA (Molecular Probes, Invitrogen) and 0.2% DMSO, the other group in vehicle control (E3+0.2% DMSO). Fish were then anaesthetized with tricaine and imaged in a 96-well-plate imaging dish by confocal microscopy. Particular attention was paid to ensure quantitative imaging in the green channel. Thirty slices were acquired through the hindbrain in the green (488/510 nm), red (547/618 nm) and DIC channels from representative fish.

### Chemical treatments

For chemical inhibition of NADPH oxidase, larvae were manually dechorionated at 36 hpf (prim25 stage) and pre-incubated for two hours in 100 µM diphenyleneiodonium (DPI) (Enzo, Farmingdale NY) containing 0.8% DMSO, or 0.8% DMSO control. After pre-incubation, larvae were infected with *C. albicans* as previously described and kept in low-melt agarose with tricaine and DPI or DMSO throughout imaging. For mortality assessment from *C. albicans* infection, larvae were removed from DPI at 4 hpi and incubated in 24-well plates in egg water at 28°C overnight. For PBS mock injection experiments, prim25 Tg*(mpx::EGFP)^i114^* larvae were dechorionated and screened by epifluorescence microscopy for abundant EGFP-expressing cells in the caudal hematopoetic tissue. Larvae were then injected with 5 nl of phosphate-buffered saline (Lonza, Inc.) or treated the same but not injected; post-injection, fish were incubated in 0.8% DMSO or 0.8% DMSO+100 µM DPI and neutrophil numbers were quantified by confocal microscopy at 4 hours post-injection. For α-tocopherol experiments, larvae were immersed in 100 µM α-tocopherol (Sigma-Aldrich, St. Louis MO) in 1% DMSO or 1% DMSO (control) from two hours pre-infection and throughout imaging. Fish were imaged from 3–6 hpi by confocal microscopy as described above. Data on GFP-Lc3 localization from all experiments were pooled and analyzed by Fisher's exact test.

## Supporting Information

Figure S1 ***C. albicans*** **does not undergo detectable levels of oxidative stress in the first four hours of infection.** 200 AB fish were injected with either Control or p47*^phox^* morpholino. 50 fish of each group were infected at the prim25 stage with OxYellow-T *C. albicans* (Control or p47*^phox^*) or with CAF2-dTomato as a control. The OxYellow-T strain has constitutive expression of dTomato from the ENO1 promoter and oxidative stress-induced expression of EGFP from the CTA1 promoter. A similar strain OxYellow, with constitutive mCherry instead of dTomato, has been previously shown to report on oxidative stress during disseminated infection in the zebrafish (Brothers et al. 2011). Fungal cells were imaged within infected fish at 4 hpi by confocal microscopy and log_2_ ratios of red and green fluorescence intensities for individual cells were quantified with ImageJ. Data shown was pooled from three independent experiments with comparable results. N = 98 with Control MO, N = 86 with p47*^phox^* MO, N = 61 for Control MO infected with CAF2-dTomato control strain.
(EPS)

Figure S2 ***C. albicans*** **does not induce detectable respiratory burst activity in recruited phagocytes.** AB zebrafish were raised to prim25 stage were either injected in the hindbrain (A, B) or not injected (C). Those injected were inoculated with 3 nL of (A) PBS+1.5×10^7^ cfu/mL CAF2-dTomato or (B) PBS. (D) As a positive control for uncaging of H_2_DCF-DA by reactive oxygen species, some fish were injected in the otic vesicle with 3 nL PBS+0.5% H_2_O_2_. Each group was divided into two and incubated in H_2_DCF-DA (500 ng/mL in 0.2% DMSO; left panels) or vehicle control (0.2% DMSO; right panels) for 1 hour. Ten fish per group were screened between one and two hours post-injection and images were acquired from representative animals. Composites include maximum projections of the red and green channels (25 slices) overlaid with a single slice in the DIC channel, or maximum projections in the red and green channels only. All images were acquired with the same settings to compare them, ensuring that the green channel detector was set just below saturation for the otic vesicle fluorescence in PBS+H_2_O_2_ injected fish. Enhancing the gain to image background fluorescence did not reveal any additional phagocytes with increased green fluorescence. In 20 fish, imaged from two independent experiments, zero cells were observed producing a detectable green fluorescence above the background level when injected with *C. albicans*. Further imaging with dihydrorhodamine gave similarly negative results in two independent experiments (data not shown). Scale bars represent 100 µm.
(TIF)

Figure S3 **Zebrafish infrequently form GFP-Lc3-positive phagosomes around** ***C. albicans*** **in an NADPH oxidase-independent manner.** (A–D) 200 Tg*(GFP-Lc3)* fish were infected with CAF2-dTomato *C. albicans* at the prim25 stage. Fish were treated with DPI or DMSO from 0 hpi or with α-tocopherol or DMSO from (−1)hpi. Fish were imaged by confocal microscopy at 3–6 hpi. (A) Examples of different types of cytosolic GFP-Lc3 localization. (B) Examples of GFP-Lc3-positive phagosomes. (C–D) Data from all experiments were pooled and analyzed by Fisher's exact test. There was no significant difference in the frequency of Lc3-positive phagosomes upon inhibition with DPI (p = 0.35) or α-tocopherol (p = 0.23) as measured by Fisher's Exact Test. Images are representative of over 1000 phagosomes imaged in 14 independent experiments. Scale bar = 10 µm.
(EPS)

Figure S4 **Confocal imaging does not notably inhibit fungal growth, filamentation, or virulence.** (A–C) CAF2-dTomato *C. albicans* were microinjected into the hindbrain ventricle of AB zebrafish and fish were screened for inoculum. One group of fish was imaged from 0 to 9 hpi, while the other was imaged only at 9 hpi. Imaging performed with 543 nm He-Ne laser at 88% power, using a 40× 0.75NA objective, with slices at 1.5 µm spacing. Pixel dwell time was 4 µs, each image dimension was 1024×1024, and total number of slices ranged from 32–40, with total active imaging time of 5–7 minutes per stack and 45–63 minutes for each fish total over the course of the experiment. (A) Image panels of a fish imaged from 0 to 9 hpi with florid fungal filamentation. Scale bar = 10 µm. (B) The number of *C. albicans* cells per fish was quantified at 9 hpi for each imaging group (N = 3) with no statistical significance (p>0.05). (C) The number of *C. albicans* filaments per fish were quantified at 9 hpi for each imaging group (n = 3) with no statistical significance (p>0.05). Error bars represent standard error of the mean. These results are representative of a large number of experiments that have been performed but were not specifically quantified. (D) Comparison of mortality in photoswitching experiments. Fish were infected and imaged as described for Fig. 1. Photoswitching was carried out as described in the Materials and Methods. Mortality was assayed at 24 hpi for photoswitched Tg(*mpeg1:GAL4/UAS:Kaede*) fish (N = 12) and non-photoswitched Tg(*mpeg1:GAL4/UAS:Kaede*) fish (N = 8). This is compared to typical 24 hpi mortality (35%) seen in this infection model for control fish in experiments described in Fig. 5 and Fig. 6 (n = 8 independent experiments).
(EPS)

Figure S5 **The effect of NADPH oxidase inhibition strictly on neutrophil and macrophage chemotaxis.** CAF2-dTomato *C. albicans* (10–20/fish) were microinjected into the hindbrain ventricle of prim25 Tg(*mpx:GFP*)^i114^ or Tg(*mpeg1:GAL4/UAS:Kaede*) fish. Immune response was monitored by time-lapse for 4 hours and the number of recruited EGFP^+^ neutrophils or Kaede^+^ macrophages were counted at 4 hpi. (A) Neutrophil recruitment in Tg(*mpx:GFP*)^i114^ control (N = 7) or p47*^phox^* (N = 8) morphants at 4 hpi. (B,C) Tg(*mpx:GFP*)^i114^ and Tg(*mpeg1:GAL4/UAS:Kaede*) fish were treated with DMSO (vehicle) or DPI from two hours prior to infection to 4 hpi. (B) The number of EGFP^+^ neutrophils in Tg(*mpx:GFP*)^i114^ fish (DMSO, N = 11; DPI, N = 12) was counted at 4 hpi (C) The number of Kaede^+^ macrophages in Tg(*mpeg1:GAL4/UAS:Kaede*) fish (DMSO, N = 15; DPI, N = 15) was counted at 4 hpi. (D) Neutrophil recruitment in Tg(*mpx:GFP*)^i114^ Control (N = 16) or duox (N = 13) morphants at 4 hpi. Numbers from fish in three independent time-lapse experiments were pooled and mean and standard error per fish are shown. ***p<0.001 by T-test.
(EPS)

Figure S6 **Percent of phagocytes that engulfed** ***C. albicans*** **.** Fish were infected at the prim25 stage and imaged by confocal microscopy at 4 hpi. The percent of fluorescent phagocytes in the hindbrain ventricle with internalized *C. albicans* were counted. (A–C) Fish were infected with CAF2-dTomato wildtype strain. (D) Fish were infected with *edt1*Δ/Δ-dTomato, *edt1*Δ/*EDT1*-dTomato, or CAF2-dTomato strains. (A–B, D) From 1 hour before infection to 4 hpi fish were treated with vehicle or DPI. (C) Control or *duox* morphants were used. (A, C, D) Tg*(mpx:EGFP)^i114^* fish were used, (B) Tg*(mpeg1:Gal4 UAS:Kaede)* fish were used. Data were compared by the non-parametric Wilcoxan test by the Chi-squared method with 2-tails, and the only significant difference seen was between Control and *duox* morphants (p = 0.02). Box plot whiskers represent the 1.5 inter-quartile range either below or above the lower or upper quartile, respectively. Data for each panel was pooled from at least three independent time-lapse experiments. Number of fish analyzed is shown below each treatment group.
(EPS)

Figure S7 **DPI treatment does not affect neutrophil recruitment to the hindbrain ventricle of mock-infected fish.** Sterile PBS was microinjected into the hindbrain ventricle of Tg*(mpx:GFP)^i114^* prim25 stage larvae. Technique was identical to that used for injection of fungi, and the needles used had the same bore and bolus size as those used for fungal injection. Fish were treated with 100 µM DPI or DMSO (vehicle) from two hours prior to microinjection to 4 hpi and imaged at 4 hpi by confocal microscopy (N = 42 and 43, respectively). Uninjected controls within the same experiment were treated with either DPI or DMSO, but were not microinjected in the hindbrain ventricle (N = 43 and 44, respectively). Data shown are means and standard errors pooled from two independent experiments with consistent results that were analyzed by Mann Whitney U tests. *p<0.05, n.s. not significant.
(EPS)

Figure S8 **In the swimbladder, DPI reduces the neutrophil migration to wounding, but not to** ***C. albicans*** **.** (A) Diagram illustrating the injection site (red line) in 4 dpf zebrafish. (B and C) 4 dpf *mpx:GFP* zebrafish were treated from 1 hour pre-injection to 4 hpi with 100 µM DPI (in DMSO) or vehicle (0.8% DMSO). Fish were injected with 5–20 yeasts of *C. albicans* CAF2-dTomato in the swimbladder. (B) The *C. albicans* inoculum was quantified by confocal microscopy within 30 minutes post-infection. (C) Neutrophils were enumerated by confocal microscopy, at the site of infection (SOI) 4 hours post infection. Four independent experiments were performed, with comparable results, and individuals from all four experiments are pooled together. Means and standard error of the mean are represented. N = 33, 30, 62, 46, 67 and 35 for the following six conditions: No injection (DMSO treated and DPI treated); PBS injection (DMSO treated and DPI treated); and Caf2-dTomato injection (DMSO treated and DPI treated), respectively. Statistical tests performed were Mann-Whitney (for panel A) and Kruskall-Wallis (for panel B), n.s. non significant, * p<0.05. (D) Representative images of 4 dpf *mpx:GFP* zebrafish 4 hpi. Images are composites of maximum projection of the red and green channels (20 slices for no injection and PBS injection and 25 slices for CAF2-dTomato injection) overlaid with a single slice in the DIC channel. Blue outlines in images define the swimbladder site of injection where the neutrophils were counted. Scale bars = 100 µm.
(EPS)
