# Supplementary figures and images for: NADPH Oxidase-Driven Phagocyte Recruitment Controls Candida albicans Filamentous Growth and Prevents Mortality
Source: PLoS Pathog. 2013 Oct 3;9(10):e1003634. doi: 10.1371/journal.ppat.1003634 (PMC3789746; doi:10.1371/journal.ppat.1003634)

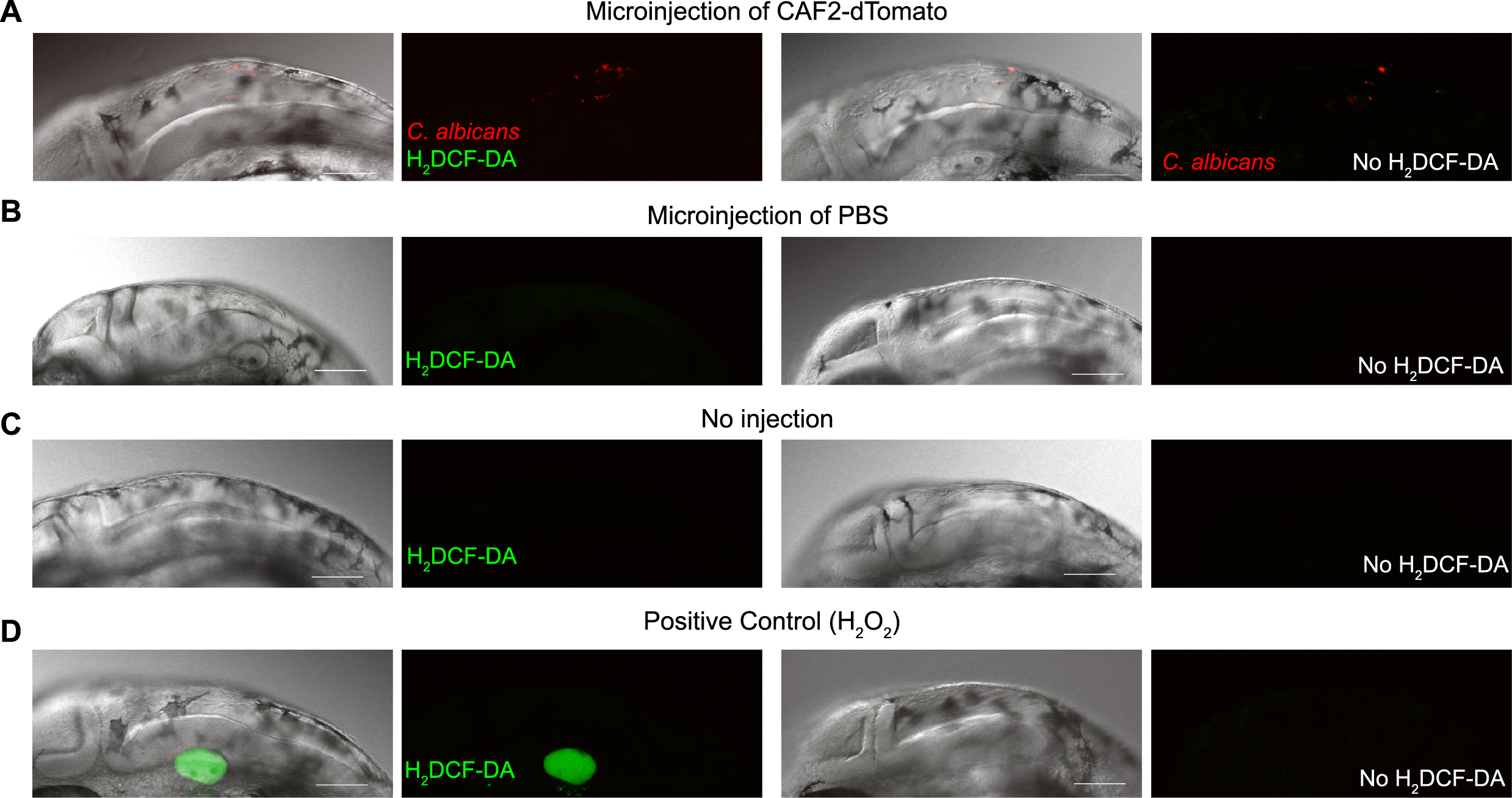

Supplement: Figure S2 — C. albicans does not induce detectable respiratory burst activity in recruited phagocytes. AB zebrafish were raised to prim25 stage were either injected in the hindbrain (A, B) or not injected (C). Those injected were inoculated with 3 nL of (A) PBS+1.5×10^7^ cfu/mL CAF2-dTomato or (B) PBS. (D) As a positive control for uncaging of H2DCF-DA by reactive oxygen species, some fish were injected in the otic vesicle with 3 nL PBS+0.5% H2O2. Each group was divided into two and incubated in H2DCF-DA (500 ng/mL in 0.2% DMSO; left panels) or vehicle control (0.2% DMSO; right panels) for 1 hour. Ten fish per group were screened between one and two hours post-injection and images were acquired from representative animals. Composites include maximum projections of the red and green channels (25 slices) overlaid with a single slice in the DIC channel, or maximum projections in the red and green channels only. All images were acquired with the same settings to compare them, ensuring that the green channel detector was set just below saturation for the otic vesicle fluorescence in PBS+H2O2 injected fish. Enhancing the gain to image background fluorescence did not reveal any additional phagocytes with increased green fluorescence. In 20 fish, imaged from two independent experiments, zero cells were observed producing a detectable green fluorescence above the background level when injected with C. albicans. Further imaging with dihydrorhodamine gave similarly negative results in two independent experiments (data not shown). Scale bars represent 100 µm. (TIF) [file ppat.1003634.s002.tif]
